# ad

**DOI:** 10.1093/geroni/igaa057.3001

**Published:** 2020-12-16

**Authors:** Justine Sefcik

**Affiliations:** Drexel University, Philadelphia, Pennsylvania, United States

## Abstract

ad

